# The Evolution of the Pulvinar Complex in Primates and Its Role in the Dorsal and Ventral Streams of Cortical Processing

**DOI:** 10.3390/vision4010003

**Published:** 2019-12-30

**Authors:** Jon H. Kaas, Mary K. L. Baldwin

**Affiliations:** 1 Department of Psychology, Vanderbilt University, Nashville, TN 37240, USA; 2 Center for Neuroscience, University of California at Davis, Davis, CA 95618, USA; mkbaldwin@ucdavis.edu

**Keywords:** superior colliculus, middle temporal area, pulvinar, primate, mammal

## Abstract

Current evidence supports the view that the visual pulvinar of primates consists of at least five nuclei, with two large nuclei, lateral pulvinar ventrolateral (PLvl) and central lateral nucleus of the inferior pulvinar (PIcl), contributing mainly to the ventral stream of cortical processing for perception, and three smaller nuclei, posterior nucleus of the inferior pulvinar (PIp), medial nucleus of the inferior pulvinar (PIm), and central medial nucleus of the inferior pulvinar (PIcm), projecting to dorsal stream visual areas for visually directed actions. In primates, both cortical streams are highly dependent on visual information distributed from primary visual cortex (V1). This area is so vital to vision that patients with V1 lesions are considered “cortically blind”. When the V1 inputs to dorsal stream area middle temporal visual area (MT) are absent, other dorsal stream areas receive visual information relayed from the superior colliculus via PIp and PIcm, thereby preserving some dorsal stream functions, a phenomenon called “blind sight”. Non-primate mammals do not have a dorsal stream area MT with V1 inputs, but superior colliculus inputs to temporal cortex can be more significant and more visual functions are preserved when V1 input is disrupted. The current review will discuss how the different visual streams, especially the dorsal stream, have changed during primate evolution and we propose which features are retained from the common ancestor of primates and their close relatives.

## 1. Introduction

This review focuses on the contributions of the visual pulvinar of primates to the two major processing streams that flow from primary visual cortex through early visual areas to targets in temporal and posterior parietal cortex. The concept of two different streams of cortical processing grew out of the results of early ablation-behavior studies on macaque monkeys and was formalized in reviews by Ungerleider and Mishkin [1] and Mishkin et al. [2]. In brief, lesions of primary visual cortex (V1 or area 17) produced severe impairments of visual behavior in monkeys and a blind spot in the visual field in humans corresponding to the extent of an incomplete lesion. Yet, lesions of inferior temporal cortex in monkeys produced deficits in recognizing qualities used for object identification, and lesions of posterior parietal cortex produced deficits in the spatial perception of objects. The different visual roles of both the inferior temporal cortex and posterior parietal cortex appeared to depend on visual inputs relayed from primary visual cortex to several secondary and higher visual areas. The two processing streams became known as the dorsal “where” pathway and the ventral “what” pathway. Subsequently, Goodale and Millner [3] advanced our understanding of the functions of the visual streams with evidence from human patients with lesions of temporal or parietal cortex. They agreed that the ventral stream is specialized for the recognition and identification of objects, but stressed the importance of the dorsal stream for providing visual guidance for motor behaviors. Thus, the dorsal stream would be considered more of a “how” than a “where” pathway. Further evidence for the relative independence of these pathways came from studies in macaque monkeys that labeled the distributions of neurons projecting to upper inferior-temporal cortex and posterior parietal cortex with different, distinguishable tracers [4,5]. The results of these two studies revealed that the visual regions projecting to these two targets are segregated to a remarkable degree, and in the small regions that do overlap, largely different neurons projected to the two targets. Consistent with other reports, Baizer et al. [4] demonstrated that the middle temporal visual area (MT) and associated areas, the medial superior temporal (MST) and the fundus of the superior temporal sulcus (FST), as parts of the MT complex [6], project to posterior parietal cortex. The projections of MT to the posterior parietal cortex (PPC) were considered to be especially significant since V1 as well as V2 and V3, project directly to the MT, and these inputs are highly dependent on relays of the information from the magnocellular layers of the lateral geniculate nucleus (LGN), while relays to the inferior temporal cortex are highly dependent on the parvocellular layers [7,8]. The magnocellular neurons relay information about visual change and location (and thus motion), and the more numerous parvocellular neurons relay information about color and visual detail (and thus about objects) [9]. It is well established that specific classes of retinal ganglion cells (RGCs) project to the different magnocellular and parvocellular cells within the LGN of primates. Thus, classes of ganglion cells of the retina are already specialized for different modes of vision, and these specializations feed into the dorsal or ventral cortical streams.

This classical view of the two cortical streams was primarily thought to depend on the functionally segregated outputs from primary visual cortex, which is heavily dependent on visual information conveyed through the above mentioned retinogeniculostriate pathways. Any relay of visual information from other extrageniculate sources, such as through the superior colliculus to cortex via the pulvinar was often neglected. Yet, an accumulation of evidence suggests that the dorsal stream of visual processing in primates emerged as an elaboration and modification of a very old system predating primates and going back to the first mammals and before. This system depended on inputs to the thalamus from the superior colliculus or its nonmammalian homolog, the optic tectum [10]. While the MT is a key cortical area in dorsal stream processing, MT is a very new visual area that emerged in early primates or their immediate ancestors [11]. However, MT may be new only in the sense that it evolved out of a region of temporal cortex by acquiring important new features. MT is easily identified by its dense myelination and characteristic retinotopic map [12] as well as direct inputs from V1 [13]. These features are not found in temporal visual areas distant from V1 of non-primate mammals. However, a relay of visual information from the superior colliculus to the pulvinar and then to temporal cortex is a very basic feature of mammalian brains, and MT could have emerged out of part of temporal cortex by changes in connections that made MT less dependent on the superior colliculus and highly dependent on newly evolved inputs from V1 (see [14] for review). This possibility is considered further here.

## 2. The Nuclei of the Primate Pulvinar and Their Connections

**Figure 1:**
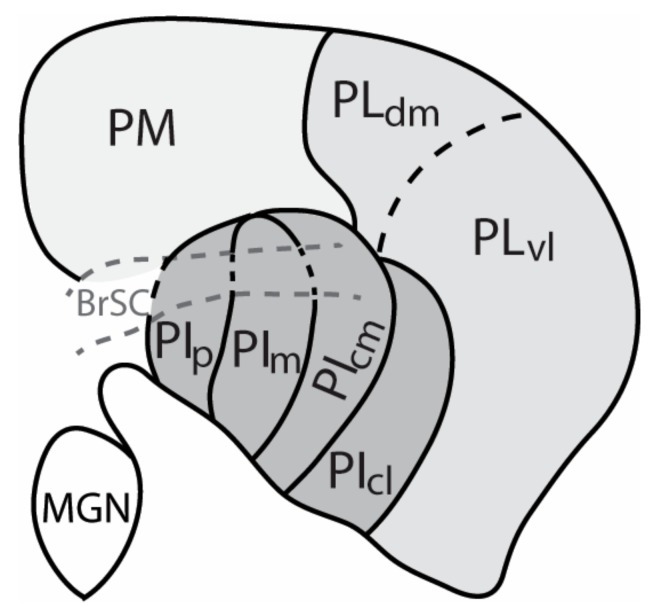
The organization and nomenclature of various divisions of the anthropoid primate pulvinar complex depicted in a coronal view of the dorsal thalamus. Different shades of grey represent the main divisions of the pulvinar nucleus: Light grey indicates the location of the medial pulvinar (PM), while medium grey: Lateral pulvinar (PL), and dark grey: Inferior pulvinar (PI) are divisions of the visual pulvinar. Multiple names and organization schemes have been proposed for the pulvinar complex (see text and [14] or review). Within the lateral pulvinar, at least two divisions, including the lateral pulvinar dorsal medial (PLdm) and lateral pulvinar ventral lateral (PLvl) divisions, have been described, while the inferior pulvinar contains four divisions: posterior nucleus of the inferior pulvinar (PIp), medial nucleus of the inferior pulvinar (PIm), central medial nucleus of the inferior pulvinar (PIcm), and central lateral nucleus of the inferior pulvinar (PIcl). PIcl, may be more aligned with PL in function based on similarities in cortical connectivity (see text). Dorsal is up and lateral is to the right.

Though modern histological procedures, and the use of different tracers to reveal connections, the pulvinar of anthropoid primates is now commonly divided into six or more nuclei (Figure 1). These include the medial pulvinar (PM) with connections that imply roles in multisensory and cognitive functions, and the nuclei of the visual pulvinar. The traditional divisions of the visual pulvinar, the lateral pulvinar (PL) and the inferior pulvinar (PI) have been subdivided in ways that are variously recognized. Even where there is good agreement on nuclei, terms for the nuclei have varied (see [14,15] for review). Here we use terms and divisions that came out of early studies on New World owl monkeys [16,17] that were subsequently modified [18,19,20]. The nucleus of the inferior pulvinar, that is densely interconnected with MT, is recognized as the medial nucleus of the inferior pulvinar (PIm). Both MT [12] and PIm [21] are retinotopically organized. The two bordering nuclei of the inferior pulvinar with dense inputs from the superior colliculus are the posterior nucleus of the inferior pulvinar (PIp) and the central medial nucleus of the inferior pulvinar (PIcm). The remaining lateral most part of the inferior pulvinar, the central lateral nucleus of the inferior pulvinar (PIcl), is larger than the other three nuclei and it contains a retinotopic map of the visual hemifield, first mapped with microelectrodes in owl monkeys [22] and subsequently in other primates. PIcm and PIcl are divisions of the central nucleus, PIc of Lin and Kaas [17], based on differences in chemoarchitecture and connections [19]. Gutierrez et al. [19] had restricted the term PIc to the smaller medial part of PIc of Lin and Kaas [17] and renamed the larger lateral part, PIl. To avoid such redefining and renaming, the divisions of PIc were called PIcm and PIcl [20]. Adams et al. [18] retained the terms used here for the nuclei of the inferior pulvinar, while subdividing the lateral pulvinar into the lateral pulvinar ventromedial (PLvm) and the lateral pulvinar ventrolateral (PLvl) divisions, in addition to a lateral pulvinar dorsal medial (PLdm) division. Most of the region defined here as PL contains a single, large retinotopic map of the contralateral visual hemifield, as shown by topographic connections with V1 and microelectrode mapping [23,24,25]. The two large nuclei with retinotopic maps, PIcl and PL, have very similar connections (see below), suggesting that they are functionally aligned. Thus, PIcl is functionally grouped with the lateral pulvinar, but renaming PIcl as part of the lateral pulvinar would come at the cost of an even more confusing range of names. As the full extent of the retinotopic map in PL is not completely clear, PL may have other nuclear subdivisions. Certainly, there is evidence for a dorsomedial subdivision, PLdm (i.e., [23,26]), and perhaps a lateral shell division of the lateral pulvinar (LS) [27] and PLvm of Adams et al. [18]. In addition, the nuclei of the pulvinar may vary across primate taxa.

**Figure 2:**
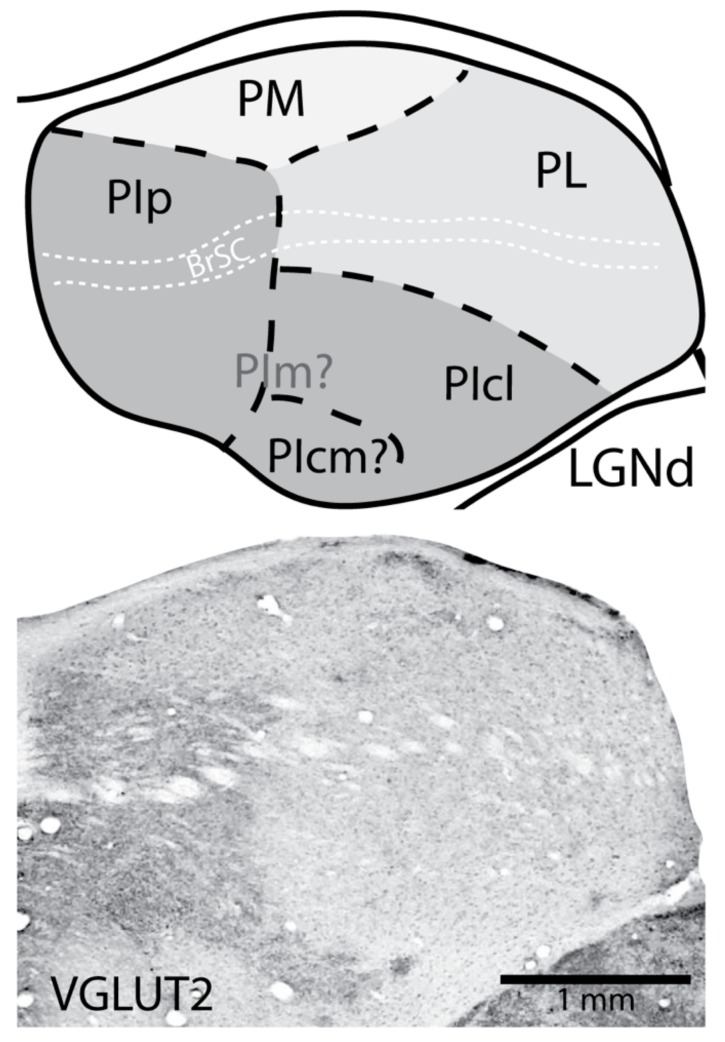
The organization of the pulvinar in galagos (a strepsirrhine primate) depicted in a coronal view of the dorsal thalamus (Top). Different shades of grey represent the main divisions of the pulvinar nucleus. Conventions are the same as those in Figure 1: Light grey indicates PM, medium grey indicates PL, and dark grey indicates PI. In galagos, the lateral pulvinar runs dorsal to the inferior pulvinar versus its lateral position in anthropoid primates (Figure 1). Further, it seems unclear where the location of the medial division of the inferior pulvinar (PIm) is located, though there are connections with the middle temporal visual area (MT), located within the region of the inferior pulvinar [36]. A clear posterior division of the inferior pulvinar (PIp) is apparent when tissue is processed for vesicular glutamate transporter 2 (VGLUT2), as is a fused smaller division which could be the homologue of central inferior division (PIcm) of the inferior pulvinar. Like anthropoid primates, the connection patterns of PIcl are more similar to those of PL than other divisions of PI. Bottom is a coronal section of the caudal aspect of the galago pulvinar processed for VGLUT2 immunohistochemistry. Dark VGLUT2 staining reveals the borders of PIp and the presumptive PIcm. Dorsal is up and lateral is to the right.

Given this background, it is also important to consider that most of what is known about the pulvinar comes from studies on a few species of New and Old World monkeys. Yet, the pulvinar complex of early primates, the ancestors of all present-day primates, diverged into several separate lines some 70–80 mya. Primates were formerly divided into two main classes, prosimians and anthropoids. Tarsiers were included with prosimians, along with galagos, lorises, and lemurs, but subsequent studies indicated that tarsiers are more closely related to anthropoids [28] Currently, many investigators use the cladistically correct terms, strepsirrhine (wet nosed) for galagos, lorises, and lemurs, and haplorrhine (dry nosed) for the clade of tarsiers, monkeys, apes, and humans. This is relevant as we consider class differences in the organizations of the visual pulvinar and the LGN. Tarsiers have architectonic divisions of the inferior pulvinar and LGN lamination patterns that match those of monkeys [29,30]. In contrast, the pulvinar of galagos, the most studied strepsirrhine primate, is somewhat different in organization compared to the pulvinar of haplorrhine primates. First, the pulvinar in galagos has rotated so that the two retinotopically organized nuclei, PL and PIcl, are adjoined as in monkeys, but PL is dorsal to PIcl rather than lateral (Figure 2). In early studies on galagos, PL and PIcl were called the dorsal and ventral pulvinar nuclei [31]. In addition, the two nuclei with dense inputs from the superior colliculus, PIp and PIcm are almost completely fused in galagos, and they might be considered to be a single nucleus, PIp, which is in the most posterior part of the pulvinar complex. The mouse lemur also appears to have a similar organization pattern as that of galagos, with dense activating inputs from the superior colliculus culminating in a single region within the posterior pulvinar [32]. The clear architectonic features of PIp and PIcm are key to defining the borders of these nuclei with PIm in New and Old World monkeys [18,19,20,33]. However, PIm is not well defined in galagos or other strepsirrhine primates [32,34,35,36], which makes defining two potentially separate divisions (PIp and PIcm) within the PIp of strepsirrhines difficult. These differences in pulvinar organization and histological features have been most informative in efforts to reconstruct the evolution of the pulvinar complex in primates [14,37].

Inferences about the functions of the nuclei of the pulvinar are perhaps best made from their types of connections. Sherman and Guillery [38] suggested that inputs to thalamic nuclei have two different functional roles. The “drivers” provide information that is transmitted to cortex. The retinal inputs to the dorsal lateral geniculate nucleus, for example, activate geniculate neurons in a powerful way that assures that the retinal information is carried on to primary visual cortex. Other inputs to the LGN, the “modulators”, do not change the message, but enhance or suppress the message so that it has more or less impact. Drivers have properties that allow them to activate target neurons with a few reliable and powerful synapses, while modulators are often from several sources and usually have relatively weak influences.

**Figure 3:**
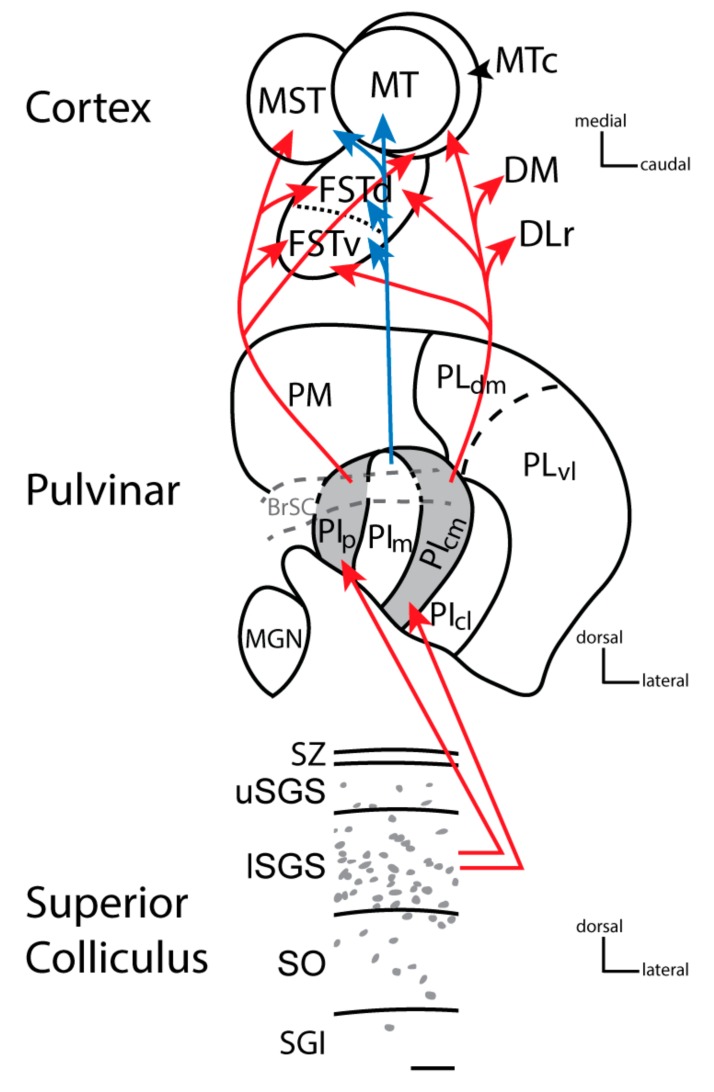
The progression of information from the superior colliculus to the pulvinar and cortex in New and Old World monkeys. Cells within the lower stratum griseum superficial (SGS) project to the posterior and central medial inferior pulvinar (see red arrows), which then project to the middle temporal complex divisions in temporal cortex, except MT itself, as well as other dorsal stream visual areas including the dorsal medial (DM) and the rostral division of the dorsal lateral (DLr) area. The cells within the superior colliculus that project to PIp and PIcm contain vesicular glutamate transporter 2 mRNA [14,33]. The terminals of these cells express VGLUT2 protein within the pulvinar resulting in PIp and PIcm staining darkly for VGLUT2 protein (grey: [14,33,51], The superior colliculus does not project to the medial division of the inferior pulvinar (PIm: [40,41,52], but PIm is this division that has strong connections with MT (see blue arrows). The image of the superior colliculus shows a coronal view of the dorsal layers of the superior colliculus: The stratum zonale (SZ), the upper stratum griseum superficiale (uSGS), the lower griseum superficiale (lSGS), the stratum opticum (SO), and the stratum griseum intermediate (SGI). Grey dots depict the general distribution of VGLUT2 mRNA within the superior colliculus in New World monkeys (Adapted from [14]. Conventions for the pulvinar are similar to Figure 1, except the dark gray shading depicts dark VGLUT2 protein staining. The cortex shows the organization of MT complex areas as they would appear in sections cut tangential to the pia surface in flattened cortex preparations (i.e., with the superior temporal sulcus opened). MT is the middle temporal area, the middle temporal crescent is MTc, FSTd and FSTv are the dorsal and ventral divisions of the fundus of the superior temporal sulcus respectively, and MST is the medial superior temporal cortex. Other divisions with connections to PIp and PIcm include the rostral division of the dorsal lateral area (DLr) and the dorsal medial area (DM). Connections with V1 [19] but see [18] and V2 [18], especially with thick stripes [53], have also been described for the medial aspect of the inferior pulvinar but are not indicated here.

**Figure 4:**
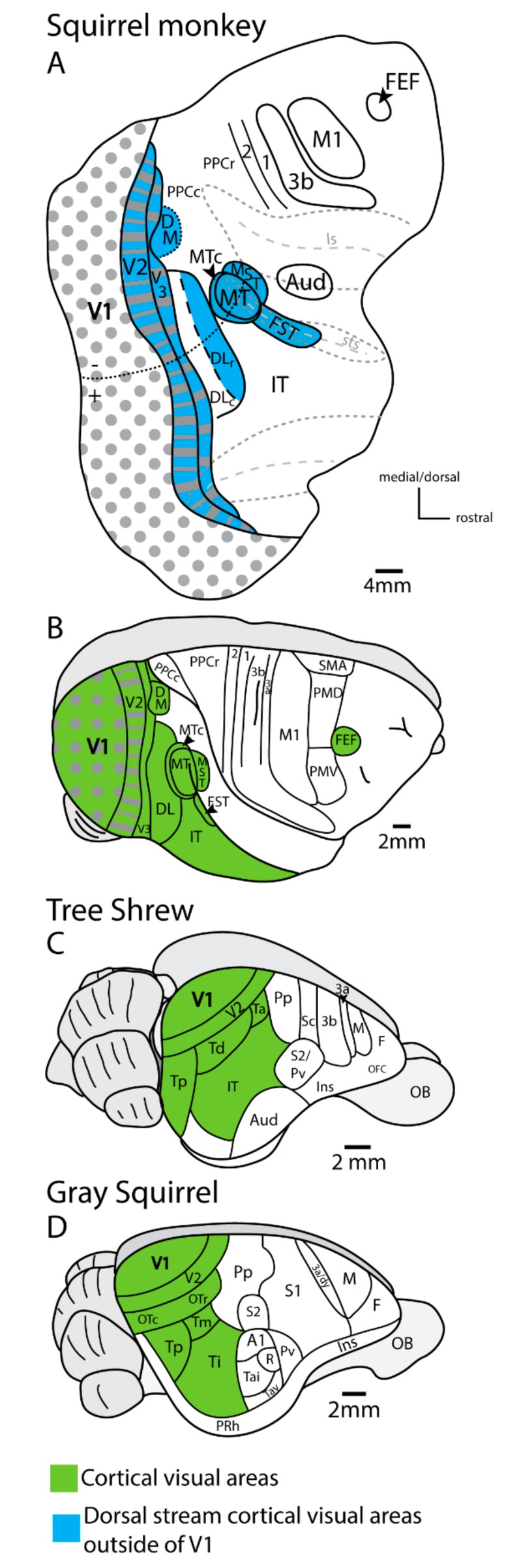
The organization of cortical visual fields in squirrel monkeys (**A**,**B**), tree shrews (**C**), and gray squirrels (**D**). (**A**) is a view of the cortical organization in a flattened view of the brain with the lateral (ls) and superior temporal sulcus (sts) opened (see Figure 3 for details on the MT complex). Cortical fields outside of V1 associated with the dorsal stream are highlighted in blue. V2 and V3 have modules that contain dorsal or ventral stream modules within them. (**B**–**D**) are lateral views of the intact cortex. For (**B**–**D**), all visual cortical fields are highlighted in green. Note that the number and complexity of cortical fields is greater in primates than their close relatives, tree shrews, and gray squirrels.

The superior colliculus sends both types of inputs to the pulvinar complex. In primates, two nuclei of the inferior pulvinar, PIp and PIcm, get driving inputs from neurons in the inner superficial gray of the superior colliculus. One of the characteristics of driving neurons in subcortical structures is that they use the vesicular glutamate transporter 2 in their terminals (e.g., [33]). The neurons in the superficial gray of the superior colliculus that project to PIp and PIcm express vesicular glutamate transporter 2 (VGLUT2) mRNA in their cell bodies and VGLUT2 protein in their synaptic boutons in PIp and PIcm [14,34,39]. PIp and PIcm then project densely to the middle layers of several areas of the MT complex (including the middle temporal crescent, MTc, the dorsal fundus of the temporal sulcus, FSTd, the ventral fundus of the temporal sulcus FSTv, and the medial superior temporal, MST, areas) (Figure 3), but very little, if at all, to MT [40,41]. The nucleus that projects to MT, PIm, has few or no inputs from the superior colliculus and is activated instead by inputs from MT. Thus, PIm is more or less a satellite of MT, receiving inputs from MT and perhaps sending back a modulating input [42], although PIm receives some direct inputs from the retina [40,43], and the neurons projecting to MT appear to be drivers by being glutamatergic and weakly parvalbumin positive [21]. In intact monkeys, MT is highly dependent on V1 for activation (e.g., [44,45]), and lesions of the superior colliculus have no notable effect on the response properties of neurons in MT [46] nor on attention effects on MT neurons [47]. On the other hand, cortex within the floor of the superior temporal sulcus including FST is a part of the MT complex which does receive superior colliculus input via the pulvinar and is functionally dependent on the superior colliculus [48]. Similar results have been reported in mice, where a lateral visual cortical area, which receives input from the superior colliculus via the pulvinar, is also dependent on superior colliculus neuronal activity [49]. These findings support the idea that this driving pathway through the caudal pulvinar from the superior colliculus has been conserved across rodents and primates and hint at a possible homologous region of cortex in rodents that has become the MT complex in primates. MT and areas of the MT complex are areas of the dorsal stream of visual cortical processing that also include the dorsomedial visual area (DM) and the rostral half of the dorsolateral visual area (DLr) or rostral V4 (Figure 4; see [50] for review).

The other nuclei of the visual pulvinar are quite different from those that predominately contribute to the dorsal stream. Most of the rest of the visual pulvinar consists of two large retinotopically organized nuclei that mirror each other, PIcl and PL (galagos: [31,54]; owl monkeys: [22]; cebus monkeys: [24]; macaque monkeys: [23,25]; humans: [45,55]. Both nuclei depend on direct and indirect inputs from V1, but not the superior colliculus, for activation [56]. In agreement, the receptive fields of neurons in these nuclei reflect those of neurons in V1 and not those of neurons in the superior colliculus [56,57]. While in primates, the superior colliculus does project to both PIcl and PL, the projections are relatively sparse and species variable [52] and they express little or no VGLUT2 in their terminals [33,34,35,51]. These inputs from the superior colliculus may have a suppressive effect on visual excitability of pulvinar neurons during eye movements [58]. Both nuclei project back to the cortical areas that project to them (V1, V2, V3, DLr, and ITc; see [59]) where they likely have a modulating influence. Consistent with a modulating role, inactivation of PL in galagos reduced or suppressed the responses of supra-granular neurons in V1 to visual stimuli [60]. The superior colliculus also projects to parts of the dorsal lateral geniculate nucleus [61] and these inputs may modulate neurons that then modulate V1 neurons and their outputs to dorsal and ventral streams [62].

## 3. The Dorsal and Ventral Streams of Visual Processing in Primates

In the early 1980s, Ungerleider and Mishkin [1] and Mishkin et al. [2] presented the exciting idea of two largely separate streams of interconnected visual areas in primates that process visual information for object identification or locations in space. The dorsal stream from primary visual cortex to posterior parietal cortex was the “where” pathway for locating objects in space and the pathway from V1 to inferior temporal cortex was the “what” pathway for object recognition. Understandings of visual cortex organization and connections were just emerging, but it was already clear that several steps across multiple visual areas were involved in both early pathways. Soon thereafter, Goodale and Milner [3] more broadly re-characterized the ventral stream as for “perception” and the dorsal stream as for “action”, or more specifically, the dorsal stream is for “on-line control of skilled actions” directed at objects [63]. These concepts have received further support by the subsequent identification of face and object sensitive domains in the temporal cortex of monkeys and humans [64,65,66] and the discovery of action specific domains in posterior parietal cortex that directly relate to functionally matched domains in primary motor cortex and premotor cortex [67,68,69,70,71].

As extensions of the dorsal stream, monkeys and prosimian galagos have small regions in the posterior parietal cortex (domains) which, when electrically stimulated, produce various complex movements or behaviors such as bringing the hand to the mouth, reaching, grasping, looking at some position in space, defending the head or body from a blow, and running or climbing (see [70] for review). These PPC domains are directly connected to functionally matched domains in M1 and premotor cortex. Selectively inactivating M1 domains abolishes the abilities of PPC domains to evoke their specific behavior [72,73]. Although the same or very similar behaviors can be evoked from PPC, PMC, and M1, functionally matched domains in these fields likely have different but complementary roles.

Kaas and Stepniewska [70] proposed that domains in each region are involved in different levels of decision-making. Domains in PPC receive mainly visual information from earlier stations in the dorsal stream of visual processing, but also higher-order somatosensory and auditory information. These sensory signals are evaluated in the PPC domains and they interact to determine what action is necessary, if any, based on the sensory information. Through the process of selective activation and mutual suppression, the most activated domain will activate matching PMC and M1 domains, where further evaluation, based largely on cingulate and prefrontal cortical inputs and thalamic inputs, may support or change the PPC selection. Subcortical outputs of domains in all these regions, but especially M1, then activate subcortical structures that directly or indirectly mediate behaviors.

A similar process occurs in inferior temporal cortex where interconnected modules, or domains, process visual information to recognize faces, individual faces as familiar or not, and objects and places as familiar or not. Thus, a face may be classified as a face or perhaps more specifically as a familiar face (e.g., [64,65,66,74]. Early recognition of the importance of the inferior temporal cortex in object perception came from single neuron recordings that revealed neurons highly responsive to hands or faces [75].

The two visual streams are highly elaborated in primates, with many areas and domains involved in all studied species of primates. In addition, both streams appear to be more elaborate in some monkeys, especially in humans [76,77]. Yet, the concept of multiple functionally segregated visual streams did not emerge with the discovery and exploration of the classical dorsal and ventral streams introduced by Ungerleider and Mishkin [1]. An earlier concept of two cortical visual systems had been proposed, mainly for non-primates [78]. In this case, the functional distinctions of the two streams were very different than those of the dorsal and ventral streams of primates: One stream was dependent on the retina-LGN-V1 pathway and the other on a retina-SC-PUL-temporal cortex pathway. However, the two cortical visual systems were not extensive, as early mammals had few cortical visual areas, little posterior parietal cortex, and few subdivisions of temporal cortex ([79]: Figure 4). Thus, we suggest that the primate dorsal stream had a forerunner in a SC-posterior pulvinar-temporal cortex pathway that has been widely retained in all mammals, but greatly augmented by direct V1 projections to MT, a key dorsal stream area. The pulvinar complex, as a whole, contributes differently to both dorsal and ventral streams. Additionally, the medial pulvinar, with widespread connections to “association” areas of cortex, contributes to the higher stations of both streams, although the specific roles of the pulvinar connections are not well understood [80].

## 4. Lesions of Primary Visual Cortex (V1) in Rodents and Tree Shrews Have Less Impact on Visual Behavior Than in Primates

As already mentioned, lesions of V1 in primates impact vision greatly, especially in humans. Though patients with V1 lesions deny seeing, they retain the ability to detect, localize, and track objects. This phenomenon has been called blindsight [81,82,83]. On the other hand, lesions of the superior colliculus in primates have little or no effect on the responsiveness of neurons in most areas of the dorsal and ventral streams of visual processing. As a notable example, lesions of the superior colliculus in macaque monkeys had “no effect” on most of the response characteristics of neurons in visual area MT [46], the visual area that is the gateway to posterior parietal areas of the dorsal stream. Removal of the superior colliculus in monkeys produces some deficits in visual attention and especially eye movements [84], but not the profound neglect that has been reported in rodents, tree shrews, and other non-primates.

In contrast, V1 lesions in tree shrews, which are closely related to primates as members of the Euarchontoglire superorder, leave many visual abilities intact (see [85] for review). After bilateral ablations of primary visual cortex, tree shrews are capable of form and pattern vision, localizing visual objects in space, following a moving food item, grasping food, and avoiding objects while running on the floor [86,87,88,89]. After lesions of the superficial gray of the superior colliculus in tree shrews, the layer that projects to the pulvinar, visual discriminations were impaired but not totally eliminated. Deeper lesions of the superior colliculus that included projections to motor centers in the brainstem, produced tree shrews that were unable to track objects or freely move about, and the tree shrews appeared to be blind [90,91]. From such evidence, Petry and Bickford [85] concluded that lesions of the superior colliculus impair vision more in tree shrews than lesions of V1.

The effect of lesions of the primary visual cortex on vision has been evaluated in several species of rodents. Rodents are less closely related to primates than tree shrews, but rodents are in the same Euarchontoglire clade. Perhaps the ablation studies on squirrels are most relevant, in that they have well developed visual systems that resemble those in tree shrews. In gray squirrels, lesions of the entire primary visual cortex did not abolish the ability to perform visual discriminations of form [92]. Lesions of primary visual cortex in ground squirrels also failed to abolish pattern vision [93]. Lesions of area 17 in less visual rats also did not disrupt the ability to discriminate visual patterns [94,95]. After removal of primary visual cortex and some of the surrounding cortex, hamsters had some difficulties with visual discriminations; while lesions of the superior colliculus abolished the ability of hamsters to orient toward a novel visual stimulus [78]. From these and other results, Schneider [78] concluded that primary visual cortex (and surrounding cortex) allows visual objects to be identified (“what is it”), while the superior colliculus is necessary in determining the locations of visual objects (“where is it?”). Thus, Schneider (and others) came to conclusions that predate current views of two cortical visual systems in primates but differed in proposing a substantial role for the superior colliculus.

We conclude from such lesion-behavior experiments that both primary visual cortex and the superior colliculus are important visual structures and that the non-primates that are close relatives of primates, and probably most non-primate mammals, are more impaired by superior colliculus lesions than are primates. This, we suggest, is because of a number of contributing factors.

A major contributing factor relates to differences in the information supplied to the superior colliculus and LGN across non-primate and primate species. For instance, nearly all retinal ganglion cells in rodents project to the superior colliculus in rodents [96,97,98], and about 80% of retinal ganglion cells project to the superior colliculus in tree shrews [99,100]. In addition, the superior colliculus in squirrels and tree shrews is much larger than the LGN, and thus provides a substantial projection to the posterior pulvinar, and then to temporal cortex (e.g., [101,102]. In contrast, only a small fraction of retinal ganglion cells project to the superior colliculus in primates (approximately 10–20%: [103]), and these RGCs are limited to the M class of cells that are specialized for stimulus contrast and change, as well as the heterogenous K cell class. Instead, the majority of RGC’s project to the LGN in primates, with roughly 80% of RGCs projecting only to the LGN [103,104,105]. As mentioned before, the cells that project to the LGN arise from functionally distinct RGCs. Three main classes of ganglion cells, the P, M, and K ganglion cells, project to the parvocellular, magnocellular, and koniocellular layers of the LGN in primates [9]. These classes correspond to the X, Y, and W classes in non-primate mammals (e.g., [106]. Especially in diurnal primates, such as macaque monkeys, the parvocellular pathway is greatly expanded [107]. These cells have small receptive fields that would be useful in object identification. This functional segregation of inputs to the LGN continues to V1 and forms the basis for the classical segregation of dorsal and ventral visual streams. Thus, for non-primates, the retino-superior colliculus-pulvinar pathways and not the retino-LGN-V1 pathways seem to provide more visual information to cortex in at least some non-primates than it does in primates.

Another contributing factor to the differences in impairment characteristics after V1 lesions in primates versus non-primates can be attributed to the further elaboration of the functionally distinct pathways from the LGN through V1 that comprise the classical dorsal and ventral streams in primates. Within V1 of primates, the terminations of magnocellular and parvocellular inputs are spatially segregated into functional modules (and distinct laminar terminations). From V1, in primates, these pathways proceed over largely different visual areas (or parts of visual areas) to different targets that mediate two quite different functions, visuomotor behavior or object identification. Thus, because the majority of RGC inputs progress through the LGN pathway in primates, lesions of V1 in primates greatly impair both dorsal and ventral stream processing. In tree shrews and rodents, as mentioned above, a greater proportion of information from the retinal is relayed through the superior colliculus, and this information flows mainly into the “dorsal stream” while the “ventral stream” of visual processing mainly depends on V1. Therefore, information associated with the dorsal stream, in these species, still predominantly relays through the superior colliculus to the posterior pulvinar and up to temporal cortex and is mostly intact after V1 lesions.

In tree shrews, the dorsal nucleus of the pulvinar (Pd) gets activating input from the superior colliculus and relays visual information to the temporal posterior division of visual cortex (TP), which projects, in turn, to other visual areas [108,109,110]. TP gets little or no direct input from V1. The direct targets of V1 include V2 and a temporal dorsal area (TD) [111], and these are the early areas of a less complicated “ventral stream”. In squirrels, the superior colliculus projects to the caudal nucleus of the pulvinar (C) [101], and this caudal nucleus projects to the temporal posterior area of cortex [112], which gets little or no direct inputs from V1 [113,114]. In contrast to primates, these mammals have separate cortical beginnings of the dorsal and ventral streams of cortical processing with the ventral stream starting in V1 and the dorsal stream starting in temporal cortex. The visual systems in other rodents, such as rats and mice, may be similar. In mice, neurons in the superficial layers of the superior colliculus that are VGLUT2 RNA positive project to the caudal pulvinar [115] which projects to temporal cortex (see [116] for review) to drive cortical neurons [49]. A similar tectal pathway to the pulvinar and then to temporal cortex has been described in rats [117,118]. In our view, this tectal pathway via the caudal pulvinar to temporal cortex represents the pre-primate primitive dorsal stream of cortical processing. This pathway was extensively described in a recent study in mice [119]. While the observations are consistent with the results from previous studies on mice noted above, the authors described the tectum to cortex pathway as “analogous but not identical to the primate visual streams”. We suggest instead that it is homologous to the tectum to posterior pulvinar to the MT complex pathway that forms part of the more complex dorsal stream of primates. It is this tectal input to cortex, we suggest, that provides blindsight to humans and other primates and sometimes much more to other mammals.

## 5. Blindsight and the Co-Evolution of PIm and MT

We have previously argued that MT is a visual area that evolved with the first primates [11,13,120]. MT as a visual area in the temporal lobe of primates that is distinguished by architectonic and functional features, but primarily by direct inputs from V1, as well as those from V2 and V3. MT is surrounded by visual areas that have connections with MT [6]; and these areas may be activated by MT. These MT complex areas (MTc, MST, FSTd, and FSTv) also have inputs from PIp and PIcm of the inferior colliculus. Both PIp and PIcm receive driving inputs from the superior colliculus and thus provide information from the superior colliculus to the MT surround, but not MT directly. PIm gets its activating inputs from MT and projects back to MT with modulating input. MT depends on V1 for activation (e.g., [44]. Both MT and PIm are unique, and somewhat puzzling as both appear as islands dependent on V1 for activation, while bordering areas or nuclei are subject to strong modulations or activation by the superior colliculus. A temporal lobe area or pulvinar nucleus with these features is not found in the relatives of primates, tree shrews or rodents, and likely any other mammals. Thus, MT and PIm emerged with the evolution of primates.

How did this happen? New structures do not appear from nothing. In the brain, new areas and nuclei likely appear by changes in connections that subdivide a nucleus or area into two or more [121,122]. Alternatively, they may result from expression of genetic factors that induce a duplication of an existing structure [12,123], but that does not seem to be the case here. Recent studies on the development of MT and PIm in marmosets by researchers in Australia led them to propose a theory that we develop further here. In marmosets, members of the Bourne laboratory reported that retinal projections to mainly PIm develop in early postnatal life, and then regress as the marmosets mature [124,125]. In related studies, Bourne and Rosa [126] report that MT matures histologically as early or nearly as early as V1. Thus, Warner et al. [125] propose that the early innervation of part of the pulvinar by the retina defines PIm, which activates MT and causes MT to mature early. This innervation of PIm by the retina may also prevent PIm from being innervated by the superior colliculus. Later in development, when the retinal inputs to PIm regress and nearly disappear, inputs from MT likely become dominant in PIm. As the activating inputs from the retina to MT via PIm regress, the activation of MT is taken over by inputs from V1.

While this theory is quite attractive, it needs further support. Retinal projections to the PIm region have been reported in other mature primates (e.g., [127,128,129]) and to the pulvinar in other mature mammals (e.g., [130,131,132,133,134]. The projections are usually sparse, and of unknown function. We do not know if retinal inputs are stronger in early development and then regress in any of these mammals. However, retinal projections appeared denser in the pulvinar of newborn compared to adult squirrels [135]. As a key factor in the overall theory, the development and possible regression of retinal projections to the inferior pulvinar needs further study. The timing of the MT territory activation by a retina-to-pulvinar-to-cortex pathway could be critical in both preventing an occupation by a superior colliculus-to-pulvinar-to cortex pathway, and the regression of the retina-to-pulvinar pathway could open up MT to domination by V1 inputs. But to what end? We suggest that the dorsal stream of visual processing in primates is a cortical system for action that pre-dates the emergence of primates and is usually present in a greatly reduced form in tree shrews, rodents, and other mammals as a stream dominated by visual information from the superior colliculus. In early mammals with little neocortex, the role of V1 in cortical processing was likely not as dominant as it is in primates where V1 is huge and much of cortex is visual. V1 clearly has more of the visual inputs from the retina via the LGN than the superior colliculus [105], and V1 has the most detailed retinotopic map (e.g., [12,136]. Overall for primates, a significant contribution of V1 to dorsal stream processing could appear to be highly beneficial. The dorsal stream of primates has access to information from V1 via MT, as well as from V1 via the dorsal medial visual area (DM) [13], and access to information from the superior colliculus via PIp and PIcm projections to MTc, FSTd, FSTv, and MST surrounding MT [50]. The benefits of these various sources of information, but especially those from V1, may be especially significant in the expansion of the dorsal stream in primates to occupy much of an expanded portion of posterior parietal cortex with direct influences on motor and premotor cortex [70]. But there appears to be a cost. Lesions of V1 in primates disable much, but not all, of dorsal stream functions. The few preserved abilities are those of blind sight in humans. More dorsal stream functions are preserved when V1 lesions occur early in postnatal life [137,138,139]. Likely, this results from removing V1 from the competition with the superior colliculus for cortical visual functions, so the roles of the visual inputs to the cortex from the inferior pulvinar can be enhanced. It appears that such inputs can more effectively activate MT after recovery from V1 lesions in early life, and more visual abilities are present in mature humans and monkeys with early damage to V1 [69,138,139]. Some reactivation of MT may occur after V1 lesions in adult monkeys and humans, possibly by new or indirect inputs from the pulvinar, and possibly the extrastriate projections from the koniocellular system in the LGN are also involved [140]. In addition, compensations for sensory loss can be greatly enhanced by only a few surviving inputs, as shown by the reactivation of somatosensory cortex and the recovery of tactile sensation after nearly complete sensory loss due to spinal cord injury [141].

## 6. Summary

The evidence considered here supports the view that the bulk of the visual pulvinar in primates consists of at least five nuclei. Three of the nuclei of the inferior pulvinar, PIp, PIm, and PIcm are critical parts of the dorsal stream of visual processing. Two of these nuclei, PIp and PIcm, receive activating inputs from the superior colliculus and relay information especially about visual motion to a collection of small visual areas (MTc, MST, FSTd, and FSTv) that surround visual area MT. Thus, these two nuclei provide visual information to the dorsal stream that is independent of dorsal stream input arising from V1. MT differs from its surrounding areas by getting direct inputs from V1, as well as from V2 and V3. These inputs are highly dependent on the magnocellular subsystem of projections to the LGN, then to V1, and then to modular divisions of V2 and V3, and all to MT. Thus, MT mainly provides information based on the magnocellular layers of the LGN to visual areas of the dorsal stream. The PIm nucleus of the inferior pulvinar is normally free of inputs from the superior colliculus. PIm is activated by inputs from MT and projects back to MT to modulate neurons activated directly or indirectly by cortical inputs. Thus, PIm can be considered a satellite of MT. The central lateral nucleus of the inferior pulvinar, PIcl, and the ventral lateral nucleus of the lateral pulvinar, PLvl, are large nuclei that are retinotopically organized, and join each other along the representation of the vertical meridian, as is similar for V1 and V2. PIcl and PL neurons project to areas V1, V2, V3, caudal DL/V4, and ITc and receive projections from these areas. These nuclei also receive modulating inputs from superior colliculus. However, PIcl and PL neurons are completely dependent on V1 for activation. Both nuclei appear to modulate neuronal responses in early visual areas, perhaps by amplifying cortical neurons in retinotopic regions exposed to visual motion. As visual areas V1, V2, V3, caudal DL/V4, and ITc are primarily concerned with ventral stream processing, PIcl and PL are largely or completely involved in ventral stream processing.

Galagos and lemurs, strepsirrhine primates, have a somewhat different arrangement of the nuclei of the pulvinar. Overall, the complex is rotated compared to that in monkeys, so that PL is dorsal to PIcl and the rest of the inferior pulvinar is posterior to these two nuclei. The activating VGLUT2 positive terminals from the superior colliculus identify a posterior nucleus that appears to correspond to PIp in anthropoid monkeys or perhaps a fused PIp plus PIcm. A VGLUT2-free sub-region corresponding to PIm does not seem to divide the VGLUT2 positive region and the PIm nucleus as distinctly as what is observed in anthropoid primates. Yet, connections with MT identify a posterior region that is likely PIm [36]. This arrangement of nuclei appears to be more primitive, because it more closely resembles the arrangement found in the pulvinar of tree shrews and rodents.

Tree shrews and rodents have a single densely labeled VGLUT2 positive nucleus that is posterior and dorsal in the complex, and projects to temporal cortex [101,142]. The VGLUT2 labeling identifies activating inputs from the superior colliculus. We regard the posterior nucleus of tree shrews and rodents, that goes by various names, as a homolog of the posterior nucleus in galagos and lemurs that divides to become PIp and PIcm. Perhaps, the nucleus should be called PIp in all these mammals. Tree shrews and rodents all appear to have one or more nuclei that are ventral and anterior to PIp and receive inputs from the superior colliculus that are less densely VGLUT2 positive [102], and may be modulating. These nuclei have interconnections with V1 and adjoining visual areas [110]. Thus, these non-primate mammals have nuclei that are likely homologous to PIcl or PL or both. However, these mammals do not have a cortical area deep into temporal cortex with inputs from V1 that could be MT, or a nucleus projecting to MT that could be PIm. These structures that are unique to primates could have evolved out of dorsal stream parts of the pulvinar and cortex that had depended on the superior colliculus for activation in non-primate ancestors.

The pulvinar of primates also receives rather sparse inputs directly from the retina. These appear to be localized within the region of the medial inferior pulvinar (where the PIp, PIm, and PIcm nuclei reside). These inputs were not always recognized in studies of retinal projections, were reported prior to the distinction of separate PIp, PIm, and PIcm divisions, and played little in theories of pulvinar organization and function. However, as a result of a series of studies on the pulvinar and visual cortex of marmosets, Bourne and collaborators have proposed a new developmental theory for the evolution of PIm and MT. In brief, the early postnatal development of a retina to PIm to MT pathway causes MT to develop nearly as early as V1, and thereby serves as a “hub” for the subsequent development of surrounding visual areas of the dorsal stream [124,125]. As the retinal projection to PIm peaks at around postnatal day 18, and then regresses, its’ functions are presumably over or greatly reduced. We suggest that the transient retinal projection to PIm may prevent innervation by the superior colliculus, and the subsequent nearly complete loss of this retina to PIm to MT pathway in later postnatal life may allow V1 inputs to MT to replace pulvinar inputs as the driving activation. These changed connections allowed PIm to emerge within the territory of PIp in the caudal pulvinar and MT to emerge in a part of temporal cortex that was part of the caudal pulvinar projection zone. The addition of V1 inputs to dorsal stream functions via MT enhanced the functions of the dorsal stream and led to an expansion of the dorsal stream projection zone in posterior parietal cortex.

Finally, as the dorsal stream of processing became more complex, and more dependent on inputs from V1 in primates, the visual impairments that occur with lesions of primary visual cortex became greater. Thus, primates, and especially humans, are more impaired after V1 lesions than tree shrews or rodents. The equivalent of human blindsight does not occur in tree shrews and rodents as much more vision is preserved. In addition, more vision is preserved in primates with lesions of V1 that are prenatal or in early infancy, suggesting that important new connections form, possibly from the superior colliculus to PIm, and transient retinal connections to the PIm may be retained [125] to compensate for the loss of V1. 

## References

[1] Ungerleider L.G., Mishkin M., Ingle D.J., Goodale M.A., Mansfield R.J.W. (1982). Two cortical visual systems. Analysis of Visual Behavior.

[2] Mishkin M., Ungerleider L.G., Macko K.A. (1983). Object vision and spatial vision: Two cortical pathways. TINS.

[3] Goodale M.A., Milner A.D. (1992). Separate visual pathways for perception and action. Trends Neurosci..

[4] Baizer J.S., Ungerleider L.G., Desimone R. (1991). Organization of visual inputs to the inferior temporal and posterior parietal cortex in macaques. J. Neurosci..

[5] Morel A., Bullier J. (1990). Anatomical segregation of two cortical visual pathways in the macaque monkey. Vis. Neurosci..

[6] Kaas J.H., Morel A. (1993). Connections of visual areas of the upper temporal lobe of owl monkeys: The MT crescent and dorsal and ventral subdivisions of FST. J. Neurosci..

[7] Livingstone M., Hubel D. (1988). Segregation of form, color, movement, and depth: Anatomy, physiology, and perception. Science.

[8] Livingstone M.S., Hubel D.H. (1987). Psychophysical evidence for separate channels for the perception of form, color, movement, and depth. J. Neurosci..

[9] Casagrande V.A., Khaytin I., Boyde J., Preuss T.M., Kaas J.H. (2007). The evolution of parallel visual pathways in the brains of primates. Evolution of the Nervous Systems.

[10] Diamond I.T., Hall W.T. (1969). Evolution of neocortex. Science.

[11] Kaas J.H., Preuss T.M., Szalay F.S., Novacek M.J., McKenna M.C. (1993). Archonten affinities as reflected in the visual system. Mammal Phylogeny; Placentals.

[12] Allman J.M., Kaas J.H. (1971). A representation of the visual field in the caudal third of the middle temporal gyrus of the owl monkey (Aotus trivirgatus). Brain Res..

[13] Krubitzer L., Kaas J. (1990). Convergence of processing channels in the extrastriate cortex of monkeys. Vis. Neurosci..

[14] Baldwin M.K.L., Balaram P., Kaas J.H. (2017). The evolution and functions of nuclei of the visual pulvinar in primates. J. Comp. Neurol..

[15] Jones E.G. (2007). The Thalamus.

[16] Lin C.S., Kaas J.H. (1980). Projections from the medial nucleus of the inferior pulvinar complex to the middle temporal area of the visual cortex. Neuroscience.

[17] Lin C.S., Kaas J.H. (1979). The inferior pulvinar complex in owl monkeys: Architectonic subdivisions and patterns of input from the superior colliculus and subdivisions of visual cortex. J. Comp. Neurol..

[18] Adams M.M., Hof P.R., Gattass R., Webster M.J., Ungerleider L.G. (2000). Visual cortical projections and chemoarchitecture of macaque monkey pulvinar. J. Comp. Neurol..

[19] Gutierrez C., Yaun A., Cusick C.G. (1995). Neurochemical subdivisions of the inferior pulvinar in macaque monkeys. J. Comp. Neurol..

[20] Stepniewska I., Kaas J.H. (1997). Architectonic subdivisions of the inferior pulvinar in New World and Old World monkeys. Vis. Neurosci..

[21] Mundinano I.C., Kwan W.C., Bourne J.A. (2019). Retinotopic specializations of cortical and thalamic inputs to area MT. Proc. Natl. Acad. Sci. USA.

[22] Allman J.M., Kaas J.H., Lane R.H., Miezin F.M. (1972). A representation of the visual field in the inferior nucleus of the pulvinar in the owl monkey (Aotus trivirgatus). Brain Res..

[23] Bender D.B. (1981). Retinotopic organization of macaque pulvinar. J. Neurophysiol..

[24] Gattass R., Oswaldo-Cruz E., Sousa A.P. (1978). Visuotopic organization of the cebus pulvinar: A double representation the contralateral hemifield. Brain Res..

[25] Ungerleider L.G., Galkin T.W., Mishkin M. (1983). Visuotopic organization of projections from striate cortex to inferior and lateral pulvinar in rhesus monkey. J. Comp. Neurol..

[26] Wilke M., Kagan I., Andersen R.A. (2013). Effects of pulvinar inactivation on spatial decision-making between equal and asymmetric reward options. J. Cogn. Neurosci..

[27] Gray D., Gutierrez C., Cusick C.G. (1999). Neurochemical organization of inferior pulvinar complex in squirrel monkeys and macaques revealed by acetylcholinesterase histochemistry, calbindin and Cat-301 immunostaining, and Wisteria floribunda agglutinin binding. J. Comp. Neurol..

[28] Jameson N.M., Hou Z.C., Sterner K.N., Weckle A., Goodman M., Steiper M.E., Wildman D.E. (2011). Genomic data reject the hypothesis of a prosimian primate clade. J. Hum. Evol..

[29] Collins C.E., Hendrickson A., Kaas J.H. (2005). Overview of the visual system of Tarsius. Anat. Rec. A Discov. Mol. Cell. Evol. Biol..

[30] Wong P., Collins C.E., Kaas J.H. (2010). Overview of Sensory Systems of *Tarsius*. Int. J. Primatol..

[31] Symonds L.L., Kaas J.H. (1978). Connections of striate cortex in the prosimian, *Galago senegalensis*. J. Comp. Neurol..

[32] Saraf M.P., Balaram P., Pifferi F., Kennedy H., Kaas J.H. (2019). The sensory thalamus and visual midbrain in mouse lemurs. J. Comp. Neurol..

[33] Balaram P., Hackett T., Kaas J.H. (2013). Differential expression of vesicular glutamate transporters 1 and 2 may identify distinct modes of glutamatergic transmission in the macaque visual system. J. Chem. Neuroanat..

[34] Balaram P., Takahata T., Kaas J.H. (2011). VGLUT2 mRNA and protein expression in the visual thalamus and midbrain of prosimian galagos (Otolemur garnetti). Eye Brain.

[35] Baldwin M.K., Balaram P., Kaas J.H. (2013). Projections of the superior colliculus to the pulvinar in prosimian galagos (Otolemur garnettii) and VGLUT2 staining of the visual pulvinar. J. Comp. Neurol..

[36] Wong P., Collins C.E., Baldwin M.K., Kaas J.H. (2009). Cortical connections of the visual pulvinar complex in prosimian galagos (Otolemur garnetti). J. Comp. Neurol..

[37] Baldwin M.K.L., Bourne J.A., Kaas J.H., Krubitzer L.A. (2017). The Evolution of Subcortical Pathways to the Extrastriate Cortex. Evolution of Nervous Systems.

[38] Sherman S.M., Guillery R.W. (2006). Exploring the Thalamus and its Role in Cortical Function.

[39] Rovo Z., Ulbert I., Acsady L. (2012). Drivers of the primate thalamus. J. Neurosci..

[40] Kwan W.C., Mundinano I.C., de Souza M.J., Lee S.C.S., Martin P.R., Grunert U., Bourne J.A. (2019). Unravelling the subcortical and retinal circuitry of the primate inferior pulvinar. J. Com. Neurol..

[41] Stepniewska I., Qi H.X., Kaas J.H. (1999). Do superior colliculus projection zones in the inferior pulvinar project to MT in primates?. Eur. J. Neurosci..

[42] Crick F., Koch C. (1998). Constraints on cortical and thalamic projections: The no-strong-loops hypothesis. Nature.

[43] Warner C.E., Goldshmit Y., Bourne J.A. (2010). Retinal afferents synapse with relay cells targeting the middle temporal area in the pulvinar and lateral geniculate nuclei. Front. Neuroanat..

[44] Collins C.E., Xu X., Khaytin I., Kaskan P.M., Casagrande V.A., Kaas J.H. (2005). Optical imaging of visually evoked responses in the middle temporal area after deactivation of primary visual cortex in adult primates. Proc. Natl. Acad. Sci. USA.

[45] Kaas J.H., Krubitzer L.A. (1992). Area 17 lesions deactivate area MT in owl monkeys. Vis. Neurosci..

[46] Rodman H.R., Gross C.G., Albright T.D. (1990). Afferent basis of visual response properties in area MT of the macaque. II. Effects of superior colliculus removal. J. Neurosci..

[47] Zenon A., Krauzlis R.J. (2012). Attention deficits without cortical neuronal deficits. Nature.

[48] Bogadhi A.R., Katz L.N., Bollimunta A., Leopold D.A., Krauzlis R.J. (2019). Midbrain activity supports high-level visual properties in primate temporal cortex. bioRxiv.

[49] Beltramo R., Scanziani M. (2019). A collicular visual cortex: Neocortical space for an ancient midbrain visual structure. Science.

[50] Kaas J.H., Lyon D.C. (2007). Pulvinar contributions to the dorsal and ventral streams of visual processing in primates. Brain Res. Rev..

[51] Baldwin M.K.L., Krubitzer L. (2018). Architectonic characteristics of the visual thalamus and superior colliculus in titi monkeys. J. Comp. Neurol..

[52] Stepniewska I., Qi H.X., Kaas J.H. (2000). Projections of the superior colliculus to subdivisions of the inferior pulvinar in New World and Old World monkeys. Vis. Neurosci..

[53] Baldwin M.K., Kaskan P.M., Zhang B., Chino Y.M., Kaas J.H. (2012). Cortical connections of V1 and V2 in early postnatal macaque monkeys. J. Comp. Neurol..

[54] Li K., Patel J., Purushothaman G., Marion R.T., Casagrande V.A. (2013). Retinotopic maps in the pulvinar of bush baby (Otolemur garnettii). J. Comp. Neurol..

[55] Arcaro M.J., Pinsk M.A., Kastner S. (2015). The Anatomical and Functional Organization of the Human Visual Pulvinar. J. Neurosci..

[56] Bender D.B. (1983). Visual activation of neurons in the primate pulvinar depends on cortex but not colliculus. Brain Res..

[57] Bender D.B. (1982). Receptive-field properties of neurons in the macaque inferior pulvinar. J. Neurophysiol..

[58] Robinson D.L., Wurtz R.H. (1976). Use of an extraretinal signal by monkey superior colliculus neurons to distinguish real from self-induced stimulus movement. J. Neurophysiol..

[59] Moore B., Li K., Kaas J.H., Liao C.C., Boal A.M., Mavity-Hudson J., Casagrande V. (2019). Cortical projections to the two retinotopic maps of primate pulvinar are distinct. J. Comp. Neurol..

[60] Purushothaman G., Marion R., Li K., Casagrande V.A. (2012). Gating and control of primary visual cortex by pulvinar. Nat. Neurosci..

[61] Harting J.K., Huerta M.F., Hashikawa T., van Lieshout D.P. (1991). Projection of the mammalian superior colliculus upon the dorsal lateral geniculate nucleus: Organization of tectogeniculate pathways in nineteen species. J. Comp. Neurol..

[62] Ahmadlou M., Zweifel L.S., Heimel J.A. (2018). Functional modulation of primary visual cortex by the superior colliculus in the mouse. Nat. Commun..

[63] Goodale M.A. (1996). Visuomotor modules in the vertebrate brain. Can. J. Physiol. Pharmacol..

[64] Moeller S., Freiwald W.A., Tsao D.Y. (2008). Patches with links: A unified system for processing faces in the macaque temporal lobe. Science.

[65] Tsao D.Y., Livingstone M.S. (2008). Mechanisms of face perception. Annu. Rev. Neurosci..

[66] Viskontas I.V., Quiroga R.Q., Fried I. (2009). Human medial temporal lobe neurons respond preferentially to personally relevant images. Proc. Natl. Acad. Sci. USA.

[67] Baldwin M.K.L., Cooke D.F., Goldring A.B., Krubitzer L. (2018). Representations of Fine Digit Movements in Posterior and Anterior Parietal Cortex Revealed Using Long-Train Intracortical Microstimulation in Macaque Monkeys. Cereb. Cortex.

[68] Cooke D.F., Taylor C.S., Moore T., Graziano M.S. (2003). Complex movements evoked by microstimulation of the ventral intraparietal area. Proc. Natl. Acad. Sci. USA.

[69] Graziano M.S., Taylor C.S., Moore T. (2002). Complex movements evoked by microstimulation of precentral cortex. Neuron.

[70] Kaas J.H., Stepniewska I. (2016). Evolution of posterior parietal cortex and parietal-frontal networks for specific actions in primates. J. Comp. Neurol..

[71] Stepniewska I., Fang P.C., Kaas J.H. (2005). Microstimulation reveals specialized subregions for different complex movements in posterior parietal cortex of prosimian galagos. Proc. Natl. Acad. Sci. USA.

[72] Cooke D.F., Stepniewska I., Miller D.J., Kaas J.H., Krubitzer L. (2015). Reversible Deactivation of Motor Cortex Reveals Functional Connectivity with Posterior Parietal Cortex in the Prosimian Galago (Otolemur garnettii). J. Neurosci..

[73] Stepniewska I., Gharbawie O.A., Burish M.J., Kaas J.H. (2014). Effects of muscimol inactivations of functional domains in motor, premotor, and posterior parietal cortex on complex movements evoked by electrical stimulation. J. Neurophysiol..

[74] Kanwisher N. (2010). Functional specificity in the human brain: A window into the functional architecture of the mind. Proc. Natl. Acad. Sci. USA.

[75] Gross C.G., Rocha-Miranda C.E., Bender D.B. (1972). Visual properties of neurons in inferotemporal cortex of the Macaque. J. Neurophysiol..

[76] Kravitz D.J., Saleem K.S., Baker C.I., Mishkin M. (2011). A new neural framework for visuospatial processing. Nat. Rev. Neurosci..

[77] Kravitz D.J., Saleem K.S., Baker C.I., Ungerleider L.G., Mishskin M. (2013). The ventral visual pathway: An expanded neural framework for the processing of object quality. Trends Cog. Sci..

[78] Schneider G.E. (1969). Two visual systems. Science.

[79] Kaas J.H., Kaas J.H., Herculano-Houzel S. (2017). The organization of neocortex in early mammals. Evolution of Nervous Systems.

[80] Homman-Ludiye J., Bourne J.A. (2019). The medial pulvinar: Function, origin and association with neurodevelopmental disorders. J. Anat..

[81] Cowey A. (2010). The blindsight saga. Exp. Brain Res..

[82] Cowey A., Stoerig P. (1997). Visual detection in monkeys with blindsight. Neuropsychologia.

[83] Stoerig P., Cowey A. (1997). Blindsight in man and monkey. Brain.

[84] Keating E.G. (1974). Impaired orientation after primate tectal lesions. Brain Res..

[85] Petry H.M., Bickford M.E. (2019). The Second Visual System of The Tree Shrew. J. Comp. Neurol..

[86] Killackey H., Snyder M., Diamond I.T. (1971). Function of striate and temporal cortex in the tree shrew. J. Comp. Physiol. Psychol..

[87] Snyder M., Diamond I.T. (1968). The organization and function of the visual cortex in tree shrews. Brain Behav. Evol..

[88] Ward J.P., Masterton B. (1970). Encephalization and visual cortex in the Tree Shrew (*Tupaia glis*). Brain Behav. Evol..

[89] Ware C.B., Casagrande V.A., Diamond I.T. (1972). Does the acuity of the tree shrew suffer from removal of striate cortex? A commentary on the paper by ward and Masterton. Brain Behav. Evol..

[90] Casagrande V.A., Harting J.K., Hall W.C., Diamond I.T., Martin G.F. (1972). Superior colliculus of the tree shrew: A structural and functional subdivision into superficial and deep layers. Science.

[91] Casagrande V.A., Diamond I.T. (1974). Ablation study of the superior colliculus in the tree shrew (*Tupaia glis*). J. Comp. Neurol..

[92] Wagor E. (1978). Pattern vision in the grey squirrel after visual cortex ablation. Behav. Biol..

[93] Levey N.H., Harris J., Jane J.A. (1973). Effects of visual cortical ablation on pattern discrimination in the ground squirrel (Citellus tridecemlineatus). Exp. Neurol..

[94] Lewellyn D., Lowes G., Isaacson R.L. (1969). Visually mediated behaviors following neocortical destruction in the rat. J. Comp. Physiol. Psychol..

[95] Mize R.R., Wetzel A.B., Thompson V.E. (1971). Contour discrimination in the rat following removal of posterior neocortex. Physiol. Behav..

[96] Chalupa L.M., Thompson I. (1980). Retinal ganglion cell projections to the superior colliculus of the hamster demonstrated by the horseradish peroxidase technique. Neurosci. Lett..

[97] Hofbauer A., Dräger U.C. (1985). Depth segregation of retinal ganglion cells projecting to mouse superior colliculus. J. Comp. Neurol..

[98] Linden R., Perry V.H. (1983). Massive retinotectal projection in rats. Brain Res..

[99] Albano J.E., Humphrey A.L., Norton T.T. (1978). Laminar organization of receptive-field properties in tree shrew superior colliculus. J. Neurophysiol..

[100] Tigges J. (1966). Ein experimenteller Beitrag zum subkortikalen optischen System von *Tupaia glis*. Folia Primat..

[101] Baldwin M.K., Wong P., Reed J.L., Kaas J.H. (2011). Superior colliculus connections with visual thalamus in gray squirrels (*Sciurus carolinensis*): Evidence for four subdivisions within the pulvinar complex. J. Comp. Neurol..

[102] Chomsung R.D., Petry H.M., Bickford M.E. (2008). Ultrastructural examination of diffuse and specific tectopulvinar projections in the tree shrew. J. Comp. Neurol..

[103] Perry V.H., Cowey A. (1984). Retinal ganglion cells that project to the superior colliculus and pretectum in the macaque monkey. Neuroscience.

[104] Perry V.H., Oehler R., Cowey A. (1984). Retinal ganglion cells that project to the dorsal lateral geniculate nucleus in the macaque monkey. Neuroscience.

[105] Weller R.E., Kaas J.H. (1989). Parameters affecting the loss of ganglion cells of the retina following ablations of striate cortex in primates. Vis. Neurosci..

[106] Sherman S.M., Wilson J.R., Kaas J.H., Webb S.V. (1976). X- and Y-cells in the dorsal lateral geniculate nucleus of the owl monkey (Aotus trivirgatus). Science.

[107] Kaas J.H., Heurta M.F., Weber J.T., Harting J.K. (1978). Patterns of retinal terminations and laminar organization of the lateral geniculate nucleus of primates. J. Comp. Neurol..

[108] Chomsung R.D., Wei H., Day-Brown J.D., Petry H.M., Bickford M.E. (2010). Synaptic organization of connections between the temporal cortex and pulvinar nucleus of the tree shrew. Cereb. Cortex.

[109] Harting J.K., Diamond I.T., Hall W.C. (1973). Anterograde degeneration study of the cortical projections of the lateral geniculate and pulvinar nuclei in the tree shrew (*Tupaia glis*). J. Comp. Neurol..

[110] Lyon D.C., Jain N., Kaas J.H. (2003). The visual pulvinar in tree shrews II. Projections of four nuclei to areas of visual cortex. J. Comp. Neurol..

[111] Sesma M.A., Casagrande V.A., Kaas J.H. (1984). Cortical connections of area 17 in tree shrews. J. Comp. Neurol..

[112] Wong P., Gharbawie O.A., Luethke L.E., Kaas J.H. (2008). Thalamic connections of architectonic subdivisions of temporal cortex in grey squirrels (*Sciurus carolinensis*). J. Comp. Neurol..

[113] Kaas J.H., Krubitzer L.A., Johanson K.L. (1989). Cortical connections of areas 17 (V-I) and 18 (V-II) of squirrels. J. Comp. Neurol..

[114] Negwer M., Liu Y.J., Schubert D., Lyon D.C. (2017). V1 connections reveal a series of elongated higher visual areas in the California ground squirrel, Otospermophilus beecheyi. J. Comp. Neurol..

[115] Gale S.D., Murphy G.J. (2018). Distinct cell types in the superficial superior colliculus project to the dorsal lateral geniculate and lateral posterior thalamic nuclei. J. Neurophysiol..

[116] Zhou N.A., Maire P.S., Masterson S.P., Bickford M.E. (2017). The mouse pulvinar nucleus: Organization of the tectorecipient zones. Vis. Neurosci..

[117] Masterson S.P., Li J., Bickford M.E. (2009). Synaptic organization of the tectorecipient zone of the rat lateral posterior nucleus. J. Comp. Neurol..

[118] Nakamura H., Hioki H., Furuta T., Kaneko T. (2015). Different cortical projections from three subdivisions of the rat lateral posterior thalamic nucleus: A single-neuron tracing study with viral vectors. Eur. J. Neurosci..

[119] Bennett C., Gale S.D., Garrett M.E., Newton M.L., Callaway E.M., Murphy G.J., Olsen S.R. (2019). Higher-order thalamic neurons channel parallel streams of visual information in mice. Neuron.

[120] Kaas J.H., Kaas J.H., Krubitzer L.A. (2006). Reconstructing the organization of neocortex of the first mammals and subsequent modifications. Evolution of Nervous Systems in Mammals.

[121] Ebbesson S.O. (1980). The parcellation theory and its relation to interspecific variability in brain organization, evolutionary and ontogenetic development, and neuronal plasticity. Cell Tissue Res..

[122] Krubitzer L. (1995). The organization of neocortex in mammals: Are species differences really so different?. Trends Neurosci..

[123] Grove E.A., Fukuchi-Shimogori T. (2003). Generating the cerebral cortical area map. Annu. Rev. Neurosci..

[124] Mundinano I.C., Fox D.M., Kwan W.C., Vidaurre D., Teo L., Homman-Ludiye J., Goodale M.A., Leopold D.A., Bourne J.A. (2018). Transient visual pathway critical for normal development of primate grasping behavior. Proc. Natl. Acad. Sci. USA.

[125] Warner C.E., Kwan W.C., Bourne J.A. (2012). The early maturation of visual cortical area MT is dependent on input from the retinorecipient medial portion of the inferior pulvinar. J. Neurosci..

[126] Bourne J.A., Rosa M.G. (2006). Hierarchical development of the primate visual cortex, as revealed by neurofilament immunoreactivity: Early maturation of the middle temporal area (MT). Cereb. Cortex.

[127] Campos-Ortega J.A., Hayhow W.R., Cluver P.F. (1970). A note on the problem of retinal projections to the inferior pulvinar nucleus of primates. Brain Res..

[128] Cowey A., Stoerig P., Bannister M. (1994). Retinal ganglion cells labelled from the pulvinar nucleus in macaque monkeys. Neuroscience.

[129] O’Brien B.J., Abel P.L., Olavarria J.F. (2001). The retinal input to calbindin-D28k-defined subdivisions in macaque inferior pulvinar. Neurosci. Lett..

[130] Kahn D.M., Krubitzer L. (2002). Retinofugal projections in the short-tailed opossum (*Monodelphis domestica*). J. Comp. Neurol..

[131] Li J., Wang S., Bickford M.E. (2003). Comparison of the ultrastructure of cortical and retinal terminals in the rat dorsal lateral geniculate and lateral posterior nuclei. J. Comp. Neurol..

[132] Major D.E., Luksch H., Karten H.J. (2000). Bottlebrush dendritic endings and large dendritic fields: Motion-detecting neurons in the mammalian tectum. J. Comp. Neurol..

[133] Ohno T., Misgeld U., Kitai S.T., Wagner A. (1975). Organization of the visual afferents into the LGd and the pulvinar of the tree shrew *Tupaia glis*. Brain Res..

[134] Somogyi G., Hajdu F., Hassler R., Wagner A. (1981). An experimental electron microscopical study of a direct retino-pulvinar pathway in the tree shrew. Exp. Brain Res..

[135] Cusick C.G., Kaas J.H. (1982). Retinal projections in adult and newborn grey squirrels. Brain Res..

[136] Lane R.H., Allman J.M., Kaas J.H., Miezin F.M. (1973). The visuotopic organization of the superior colliculus of the owl monkey (Aotus trivirgatus) and the bush baby (*Galago senegalensis*). Brain Res..

[137] Gross C.G., Moore T., Rodman H.R. (2004). Visually guided behavior after V1 lesions in young and adult monkeys and its relation to blindsight in humans. Prog. Brain Res..

[138] Moore T., Rodman H.R., Repp A.B., Gross C.G., Mezrich R.S. (1996). Greater residual vision in monkeys after striate cortex damage in infancy. J. Neurophysiol..

[139] Warner C.E., Kwan W.C., Wright D., Johnston L.A., Egan G.F., Bourne J.A. (2015). Preservation of vision by the pulvinar following early-life primary visual cortex lesions. Curr. Biol..

[140] Schmid M.C., Mrowka S.W., Turchi J., Saunders R.C., Wilke M., Peters A.J., Ye F.Q., Leopold D.A. (2010). Blindsight depends on the lateral geniculate nucleus. Nature.

[141] Liao C.C., Reed J.L., Qi H.X., Sawyer E.K., Kaas J.H. (2018). Second-order spinal cord pathway contributes to cortical responses after long recoveries from dorsal column injury in squirrel monkeys. Proc. Natl. Acad. Sci. USA.

[142] Balaram P., Isaamullah M., Petry H.M., Bickford M.E., Kaas J.H. (2015). Distributions of vesicular glutamate transporters 1 and 2 in the visual system of tree shrews (*Tupaia belangeri*). J. Comp. Neurol..

